# Propagation, Establishment, and Early Fruit Production of Table Grape Microvines in an LED‐Lit Hydroponics System: A Demonstration Case Study

**DOI:** 10.1002/pei3.70018

**Published:** 2024-12-01

**Authors:** E. S. Malai, C. A. O'Sullivan, T. J. Grant, L. Sreekantan, V. A. Mellor, S. Schmidt, I. B. Dry

**Affiliations:** ^1^ CSIRO Agriculture and Food St Lucia Queensland Australia; ^2^ School of Agriculture and Food Sciences The University of Queensland Brisbane Queensland Australia; ^3^ Centre of Excellence in Agri‐Food Systems and Nutrition of Eduardo Mondlane University (CE‐AFSN) Maputo Mozambique; ^4^ CSIRO Agriculture and Food Glen Osmond South Australia Australia

**Keywords:** CEF, controlled environment agriculture, horticulture breeding, indoor farm, protected cropping, urban farming, vertical farming

## Abstract

Controlled environment farming (CEF) systems, including tunnel houses, glasshouses, and vertical farms, are expanding worldwide. As the industry scales, growers need a broader range of crops that are adapted to CEF systems to take full advantage of the potential to increase yields and decrease weather‐related risks. Dwarf grapevines (microvines) are ideal candidates for CEF due to their high economic value, phenotype, and phenology. This study aimed to develop propagation protocols, a critical first step for the successful integration of microvines in the CEF market, and to demonstrate the establishment, early growth, first flowering, and fruiting of table grape microvines in a fully indoor, LED‐lit, CEF system. An experiment was conducted to investigate the efficiency of clonal propagation of a newly developed microvine variety, which had been bred for the production of seedless table grapes in response to two variables: (a) shoot position of cutting, and (b) length of time of misting exposure (from 3 to 7 weeks). A subset of successfully established plantlets were then transplanted into a hydroponic, CEF system, where their establishment, early growth, flowering, and fruit formation were assessed. Three weeks after cuttings were taken, 83.7% of the cuttings had formed roots, regardless of cutting section or misting treatment, while the remaining 16.7% of cuttings died. The sprouting success was lower with 49.3% of plants forming new leaves after 7 weeks. The highest level of sprouting was observed with cuttings taken from mid‐shoot and lower shoot positions and the 5‐week misting duration. While the rooting efficiency and survival of green shoot microvine cuttings are very high, further research is needed to increase the frequency of sprouting in the required timeframes to levels that are more acceptable for commercial production. The establishment success of sprouted cuttings after transplanting to hydroponics was 100% and their production and fruit quality were similar regardless of cutting tissue source. The crop cycle from planting to first harvest was 208 days (63 days for plantlet production and 145 days from transplanting to first harvest). The vines began flowering after an average of 33.9 days and the berries went through veraison (i.e., commencement of ripening) after an average of 116 days under the conditions tested. Microvine fruit grown under these conditions contained greater than the minimum total soluble solids content required for the Australian market. We have demonstrated that table grape microvines have potential as a novel crop for CEF systems.

## Graphical abstract

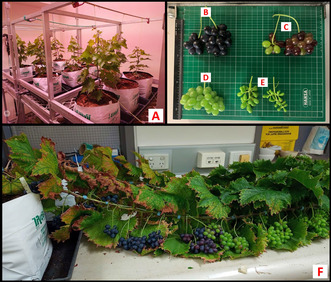

## 1 Introduction

Investment in controlled environment farming (CEF) systems is increasing worldwide (Chavan et al. 2022; Cowan et al. 2022; O'Sullivan et al. 2020). CEF systems vary in the level of technology employed and the amount of environmental control offered, from high‐tech indoor vertical farms and glasshouses, through to lower‐tech polytunnels and shade structures (Goldstein et al. 2016). The demand is driven by the need to intensify production in the face of an increasingly variable climate, growing global populations, urbanization, and constraints on the availability of productive agricultural land (United Nations 2019; Van Vliet, Eitelberg, and Verburg 2017; Hatfield, Sauer, and Cruse 2017; Campbell et al. 2017). In parallel, there is an increasing demand for pesticide‐free and locally produced food that causes minimum environmental harm (Van Delden et al. 2021).

The decreasing capacity of soil‐based food systems to deliver the volume of high‐quality, low‐input, food required by growing urban populations is increasing interest in soil‐independent production systems (Beacham, Vickers, and Monaghan 2019). CEF systems allow farmers to increase yield per unit area, extend their growing and harvesting windows, decrease water, and agrochemical inputs and escape adverse weather (Eigenbrod and Gruda 2015; O'Sullivan et al. 2020; Van Delden et al. 2021). However, despite the potential, the contribution of CEF to global food production remains small, accounting for a mere 5%–10% of global vegetable, tuber, and legume production (Clinton et al. 2018).

There are several issues that CEF production systems need to address as the industry scales globally. Key barriers are the high capital cost of establishment, and the high energy and labor costs of operation However, another limitation at present is the lack of diversity of crops that can be grown in CEF (O'Sullivan et al. 2020; Van Delden et al. 2021; Chavan et al. 2022).

Currently, CEF farms are dominated by a small range of adapted crops, including leafy greens, herbs, and vine crops, especially lettuce, peppers, strawberries, and tomatoes (O'Sullivan et al. 2020; Artemis 2020). Expanding the application of CEF to a broader range of crops, including fruiting crops, and making intensive CEF production more affordable with lower‐cost systems, would enable their full potential (O'Sullivan et al. 2019). Adapting crops to CEF can be achieved through breeding or genetic manipulation targeting plant architecture and phenology or by harnessing naturally occurring mutations that result in traits such as smaller size, shorter production cycles, and higher harvest index (Kwon et al. 2020). Traits that are advantageous for CEF farms differ from those required for outdoor production. Field crops are bred to maximize their yield in the face of a range of abiotic (heat, frost, drought, etc.) and biotic (pest and diseases) stresses. In contrast, plants grown in CEF do not need to be as hardy because their growing environment is controlled, but they need to be high value, have small stature, short times to harvest and, ideally, desirable consumer traits like unique flavors or appearance (O'Sullivan et al. 2019). For established CEF crops (leafy greens, tomatoes, berries, capsicum, cucumber, etc.) there are existing cultivars that enable this transition. For other crops, significant adaptations to plant morphology and life cycle are needed to make soilless, indoor production viable.

Grape ( *Vitis vinifera* L.) microvines, dwarf mutants of grapes, are ideal candidates for CEF due to their economic value, phenotype, and phenology. With ~25 million tons of table grapes produced annually (United States Department of Agriculture 2021), grapes account for nearly 10% of global fruit production as the fourth major fruit crop after bananas, watermelons, and apples (Shahbandeh 2022), with a global market value of ~$USD 51 billion (Research and Markets 2021). Traditional grapevines are long‐lived perennials, which take several years to reach full commercial productivity and produce high‐value fruit over a period of decades once established (Deng et al. 2016). However, current field production methods are likely to be impacted by climate change‐related increases in abiotic and biotic stresses (Webb, Whetton, and Barlow 2007; Palliotti et al. 2014). CEF offers protection from these stresses while also enabling year‐round production across a wide variety of environments, and the potential to shorten supply chains by growing fruit closer to markets (O'Sullivan et al. 2019; Benke and Tomkins 2017).

Growth of grapevines in hydroponic CEF systems has been reported in the literature; however, these studies have been focused on addressing specific research questions (e.g., rootstock development, or plant nutrition) rather than exploring CEF as a production system suitable for grapes (Yildirim et al. 2016; Chenenaoui, Daldoul, and Mliki 2017; Buoso et al. 2020; Juang et al. 2012; Daskalakis et al. 2018). While it is possible to grow conventional field grapevine cultivars in hydroponics, they are not adapted to commercial production under CEF due to their large size, long establishment times, and long juvenile phase.

Microvines were originally recovered from the wine grape cultivar, Pinot Meunier, by somatic embryogenesis and are the result of a somatic mutation in the *VvGai1* gene, which is involved in gibberellin signaling (Boss and Thomas 2002). In grapes, gibberellins stimulate vegetative growth and inhibit the conversion of tendrils into inflorescences (Coombe 1967). The absence of gibberellin signaling provides the microvine with desirable traits for CEF including dwarf stature due to shorter internodes, tendrils converted into inflorescences, meaning that fruit bunches form at every internode not just the terminal node, a short juvenile phase and rapid flowering and ripening, leading to a short cycle of 5–6 months from planting to first fruit maturation, and indeterminate growth and continuous flowering and fruiting after the onset of flowering (Torregrosa et al. 2019).

To date, the microvine has been used predominately as a model plant for research into grapevine physiology and genetics (Rienth et al. 2012; Sanchez‐Gomez et al. 2018; Dias et al. 2019). It has also been incorporated into marker‐assisted grapevine breeding programs for the production of new winegrape varieties with resistance to powdery and downy mildew (Dry and Thomas 2015; Dry et al. 2022). More recently, the potential for microvines to be grown as a commercial CEF crop for table grape production has been explored (Anon 2020; CSIRO 2022). To do this, the microvine was successfully crossed with table grape varieties to produce seedless grapes with a range of colors (black, red, and white), flavors, and textures. In addition, the *Run1*/*Rpv1* resistance locus from the wild North American grapevine species *Muscadinia rotundifolia* (Dry et al. 2022) has been introgressed so that these table grape microvine varieties are resistant to downy mildew and powdery mildew and, thus, can be grown with much lower chemical inputs than traditional table grape varieties. However, there remains a knowledge gap regarding how microvines should be managed under CEF to achieve commercially viable yields and meet consumer preferences for fruit quality.

This study demonstrates the potential of using microvines for the production of table grapes under controlled environment conditions in a system configuration similar to that used for the production of vine tomatoes in CEF. The experiment focused on the development of propagation and transplantation protocols, and establishment of the vine up to production of first fruit, critical first steps for proving that microvine crops can function in CEF. The results will assist in the design of microvine propagation and establishment protocols and identify potential areas for further research to maximize the success rate of microvine clonal propagation and CEF growth.

## 2 Materials and Methods

### 2.1 Cutting Material and Growing Environment

The parent microvines that were used to collect cutting material for this study were developed by CSIRO at the Waite Campus, Adelaide, through a breeding program that crossed microvine breeding lines with commercially available table grape varieties with desirable traits (e.g., seedlessness, large berry size, good flavor). The resulting lines were selected for dwarf stature, short juvenile stage, continuous, and precocious flowering under glasshouse conditions, and desirable fruit qualities for the table‐grape market. The microvine line used in this study (laboratory ID number 81) is a seedless, black, powdery, and downy mildew‐resistant microvine derived from a cross between a CSIRO breeding line and the table grape cultivar, Crimson Seedless. Parents were clonally propagated to produce 12 plants from which cuttings were taken. Cuttings were collected from 10‐month‐old potted vines that were producing fruit. All bunches were removed from shoots prior to collecting cuttings.

### 2.2 Propagation Experiment

**FIGURE 1:**
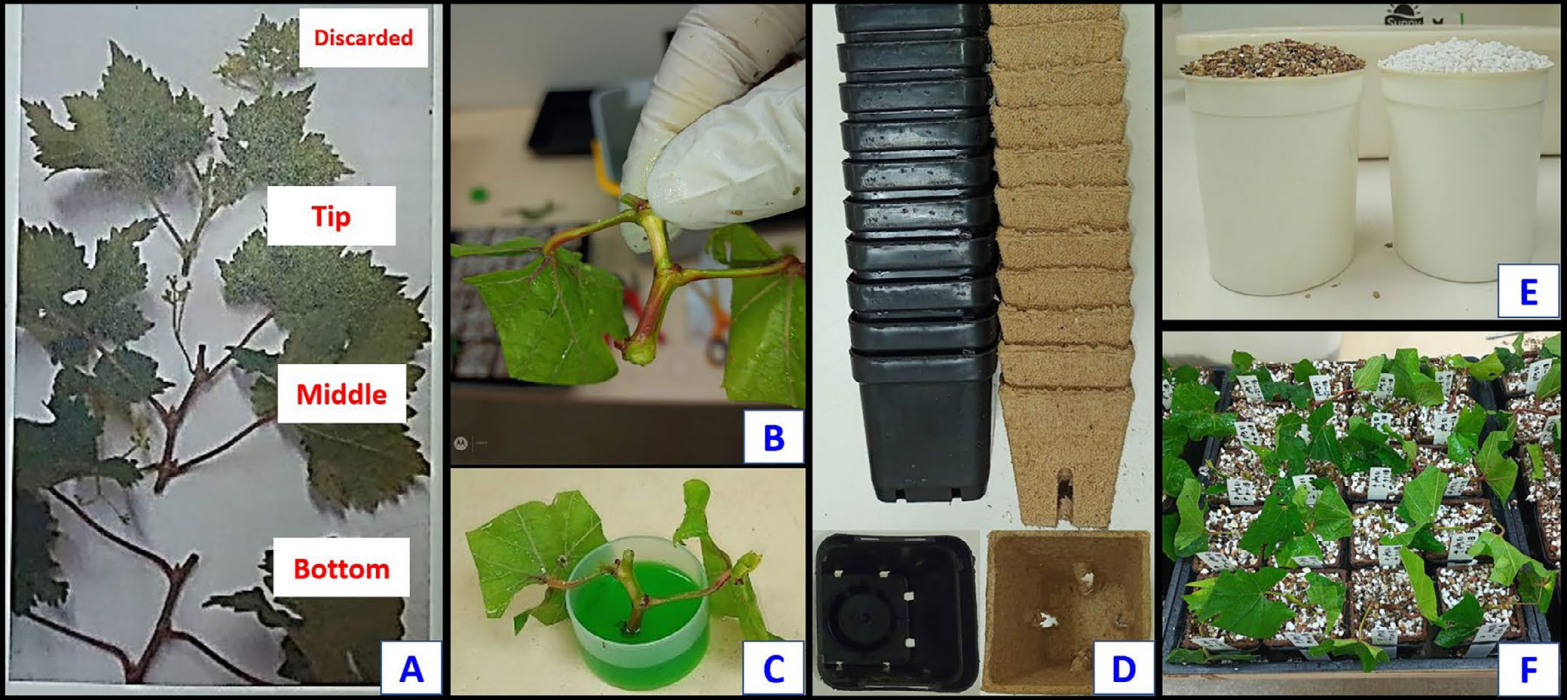
Division of microvine branches into three‐nodded cuttings (A), leaf trimming (B), dipping cuttings in Indole‐3‐butyric acid solution (C), squat pots (left) and Jiffy pots (right) (D), 50:50 vermiculite and perlite (E) and cuttings planted in the pots (F).

The propagation experiment investigated the effect of the cutting position on the shoot, and the duration of overhead misting on rooting, bud sprouting, and cutting survival. The experimental design was completely randomized with 10 replicates for each treatment (one cutting/replicate). The treatments derived from a complete factorial design consisting of two factors: five misting duration treatments (3, 4, 5, 6, and 7 weeks after cuttings were taken) and three different cutting tissue types (shoot tip, middle, and lower cane sections, Figure 1A), resulting in 15 treatments (Table 1).

**TABLE 1:** Propagation experiment treatments.

Cutting section	Mist exposure				
	3 weeks	4 weeks	5 weeks	6 weeks	7 weeks
Tip (1–3)	T1	T2	T3	T4	T5
Middle (4–6)	T6	T7	T8	T9	T10
Lower (7–9)	T11	T12	T13	T14	T15

#### 2.2.1 Cutting Collection and Planting

Healthy, green shoots (not lignified) with at least 12 nodes (counted from the shoot tip downwards) were collected and divided into three‐node sections starting from the tip to the bottom (Figure 1A). All cutting tools were sterilized with 80% ethanol to prevent the potential spread of diseases (Waite, Whitelaw‐Weckert, and Torley 2015). The first (apical) cutting was discarded as it was too fragile and would likely fail to root and sprout. The remaining three‐node cuttings (from tip to base) were designated as tip (nodes 1–3), middle/mid (nodes 4–6), and bottom/lower (nodes 7–9) cutting sections (Figure 1A; Table 1).

The bottom leaf of each cutting was detached, and the bottom axillary bud was cut at an angle through the node (Figure 1B) to promote better rooting. The remaining leaves were trimmed to a final size of approx. 2–3 cm^2^ (Figure 1B) to reduce transpiration and prevent excessive cutting dehydration while retaining enough leaf area to enable photosynthesis to continue to supply carbohydrates for the development of roots and shoots (Noyce et al. 2022). The base of the cuttings was dipped into Clonex rooting hormone gel green (Growth Technology, WA, Australia) containing the rooting hormone IBA (Indole‐3‐butyric acid) at a concentration of 1.5 g.L^−1^ for around 10 s (Figure 1C) to stimulate root production (Noyce et al. 2022). The treated cuttings were then planted into a 50:50 mix of perlite and vermiculite in 8 mm × 8 mm peat pots (Jiffy Pots, Netherlands) (Figure 1D–F).

**FIGURE 2:**
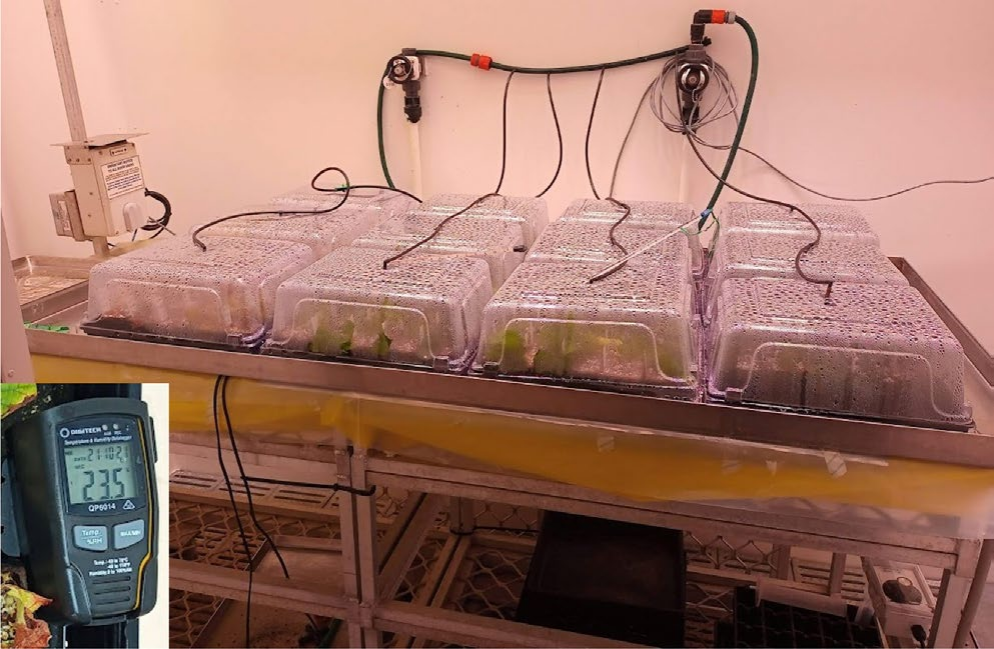
Microvine cuttings in the misting chamber and heating mat.

After planting, the cuttings were placed into misting chambers to prevent dehydration during rooting (Figure 2), and the chambers were placed into a controlled environment growth chamber set to 18°C night and 26°C day temperatures and ambient CO_2_ concentration. The misting chambers were placed on a heating mat set to a temperature of 30°C to provide bottom heat to encourage root formation (Hartmann et al. 2013). The light source in the grow room used for the propagation experiment was made up of 3 × 1000 W metal halide Lamps and 3 × 660 W Heliospectra E602 4 Band Luminaires, providing mixed, broad spectrum, white light. The light intensity in the growth chamber was set to 450 μmol.m^−2^.s^−1^ with a 16‐h photoperiod. The irrigation was automated and provided through overhead misting, occurring at 6‐h intervals for 5 s. The cuttings were kept in the misting chamber for up to 7 weeks, following the treatments described in Table 1. Cuttings that were subjected to shorter misting time treatments were removed from the misting chambers at their allocated timings but kept in the same growth room and watered by a dripper irrigation system until the end of the 7‐week propagation experiment. A data logger was used to monitor temperature and relative humidity variations within the misting chambers.

Before planting, the stem diameter of each cutting section was measured at the mid‐point using an analog caliper. The number of cuttings that rooted, sprouted, and survived was monitored weekly, starting 3 weeks after planting (3‐week misting data). Cuttings were considered rooted when they presented at least one adventitious root, while buds were considered sprouted when at least one new leaf was observed. At the end of the experiments, the data were used to calculate the percentage of rooting, sprouting, and survival.

At the end of the propagation experiment (7 weeks after cuttings were taken), a subset of plants (15 plantlets) were selected for the transplantation and early growth experiment described below.

### 2.3 Transplantation and Early Growth Demonstration: Assessing Transplantation, Establishment, Time to Flowering, and Fruit Formation

The transplantation and early growth trial were designed to demonstrate the establishment and early growth of microvines in a fully indoor, hydroponic system and to assess the impact of cutting tissue source (tip, middle, or bottom) on the transplantation success, establishment, and growth of the young vines. The experimental design was completely randomized with the cutting section as the single factor (three treatments: tip, middle, and lower sections) and five replicates per treatment (one plantlet per replicate).

#### 2.3.1 Transplantation and Fertigation Management of Vines

Five cuttings from each cutting tissue source were randomly selected from the successfully budded plantlets produced in the propagation experiment. The plantlets were healthy and vigorous (Figure 4C,D), and at the same development stage. The plantlets were transplanted, with minimum disturbance to the roots, to the center of 8.0 L coconut coir‐filled plastic bags (8 L Easyfil Planter Bag, Galuku International Pty Ltd. NSW, Australia).

Plants were placed into a growth chamber set to day/night temperatures of 26°C/20°C, 70% humidity, and ambient CO_2_. The light source in the grow room used for the establishment and growth experiment was the same mix of lights used for the propagation experiment but with 6 × 1000 W Metal Halide Lamps and 6 × 660 W Heliospectra E602 4 Band Luminaires, because the area of the growth room was double in size. The photoperiod was 16 h with a light intensity measurement at the canopy level of approximately 550 μmol.m^−2^.s^−1^ at transplanting but light intensity increased as plants gained height, reaching approximately 1150 μmol.m^−2^.s^−1^ on the day of harvest.

Plants were irrigated with drippers and nutrients supplied by fertigation without recirculation. For the first 3 days, the plantlets were only irrigated with water. From the fourth until the eighth day, the plants were fertigated with a low nutrient concentration solution (GT Ionic Grow, Hydroponic Solutions, Perth, Australia), with an electric conductivity (EC) around 0.2–0.3 mS and pH 5.8–6.0. From Day 9 onwards, a higher concentration nutrient solution (Uni‐One Uber, Uber Nutrients) was applied, with the EC maintained between 0.6 and 1.2 mS and the pH between 5.8 and 6. The nutrient solution macro and micronutrient composition and concentration are presented in Table 2. The fertigation was intermittent and set to six equally spaced events during the light phase (16‐h photoperiod). The volume of solution applied increased over time, and by the harvesting day, it was around 430 mL.plant^−1^.day^−1^.

**TABLE 2:** Nutrient solution composition and concentration (%weight/volume).

Nutrient	GT ionic grow (%*w*/*v*)	Uni‐one uber (%*w*/*v*)
Nitrate‐nitrogen	2.12	4.0
Ammonium‐nitrogen	0.18	0.0
Phosphorus	2.3	3.0
Potassium	0.33	6.0
Calcium	2.89	2.0
Magnesium	0.95	1.0
Sulfur	0.42	0.4
Iron	0.11	0.04
Manganese	0.03	0.016
Boron	0.01	0.006
Zinc	0.003	0.016
Copper	0.002	0.0016
Molybdenum	0.0005	0.0017

The plants were trained to grow vertically on guide wires, with all secondary branches pruned weekly to optimize growth and yield on the main stem. No plant growth regulators, other than Clonex root stimulator, were applied in the experiment.

Vegetative and reproductive growth data were collected weekly until the vines were harvested. Destructive harvesting of the vines occurred when the first bunches of fruit were fully ripened, but it should be noted that this is still early in the life cycle of these vines. Vegetative growth was tracked by counting the number of nodes and measuring the main stem length using a ruler. The data were used to estimate the time points when an increase in total vine length and growth rate started to occur.

For the reproductive growth, the main data collected were the number of inflorescences and fruit clusters, the timing of bloom, and veraison onsets. The time to the onset of flowering was recorded as the time (days after transplanting) required for 50% of the plants to produce at least one inflorescence and time to onset of veraison was recorded as the time (days after transplanting) required for 50% of the plants in each treatment to produce at least one fruit cluster undergoing color change (veraison).

Fruit was harvested 145 days after transplanting and consisted of collecting all the fruits fully ripened or ripening (Figure 6B,C). These fruits were weighed to estimate the yield per plant and the total yield. Other measures of productivity were berry counts per cluster for the five most mature clusters on the harvest day, plus average berry weight (calculated from the average weight of 10 berries) and cluster counts per vine. Fruit quality was assessed by measuring pH, and total acidity using FTIR (Fourier transform infrared spectroscopy) on an OenoFoss analyzer following the protocols described by Dambergs, Gishen, and Cozzolino (2015), from the five first bunches counting from the bottom of the vines (most mature). Total soluble sugar (^o^Brix) was measured by a hand‐held refractometer.

### 2.4 Data Analysis

#### 2.4.1 Propagation Experiment

A generalized linear model with a Binomial error variance was used to assess the likelihood of sprouting and survival of the cuttings as affected by the cutting section and misting treatments. A pairwise post hoc test was used to compare sprouting and survival rates under the different treatments.

#### 2.4.2 Transplantation and Early Growth Trial

A broken stick (segmented regression) model was used to assess the effect of cutting sections on the timing of changes in vine growth rate (new node formation and stem elongation), blooming onset, fruit cluster formation, and ripening onset. The models' coefficients were compared using confidence intervals to determine the significance of treatments (significant effects *p* < 0.05), while Fisher's least significant difference (LSD) test was used to compare the mean model intercepts, transition points, and rates of increase.

ANOVA was used to analyze all other variables, considering the misting and/or cutting section as factors, depending on the experiment, with a pairwise post hoc test used for mean comparisons. A principal component analysis and Pearson's pairwise correlation were performed to assess the correlation between maturation and other oenological parameters.

The significance level considered for all analyses was 5%. Statistical analyses were conducted in R with emmeans and multcomp packages (Hothorn, Bretz, and Westfall 2008; Lenth et al. 2023; R Core Team 2023).

## 3 Results

This study investigated the clonal propagation efficiency of microvines in response to different treatments and assessed the transplantation success, and early growth of the resulting plantlets through two experiments: (i) a propagation experiment to assess the effects of cutting tissue‐type and misting on rooting, sprouting and survival of cuttings from microvine parents; (ii) a growth experiment to assess the establishment, growth rate and time to first harvest of microvine plantlets, in an indoor, hydroponic growing system.

The main hypothesis for both experiments was that the shoot cutting tissue type (tip, mid cane, or lower cane) affects the clonal propagation success rate and growth of microvine cuttings, as found in previous propagation studies on other species (Daskalakis et al. 2018; Hansen 1986; Neves, Souza, and De Oliveira 2020).

### 3.1 Propagation Experiment

The temperature and humidity conditions for the propagation experiment were measured under the plastic domes to assess the conditions experienced by the plants. The average temperature under the domes varied from 15°C to 32°C and 40% and 99% with an average of 85% (Figure S1). The light intensity in the growth room was 450 μmol.m^−2^.s^−1^.

**FIGURE 3:**
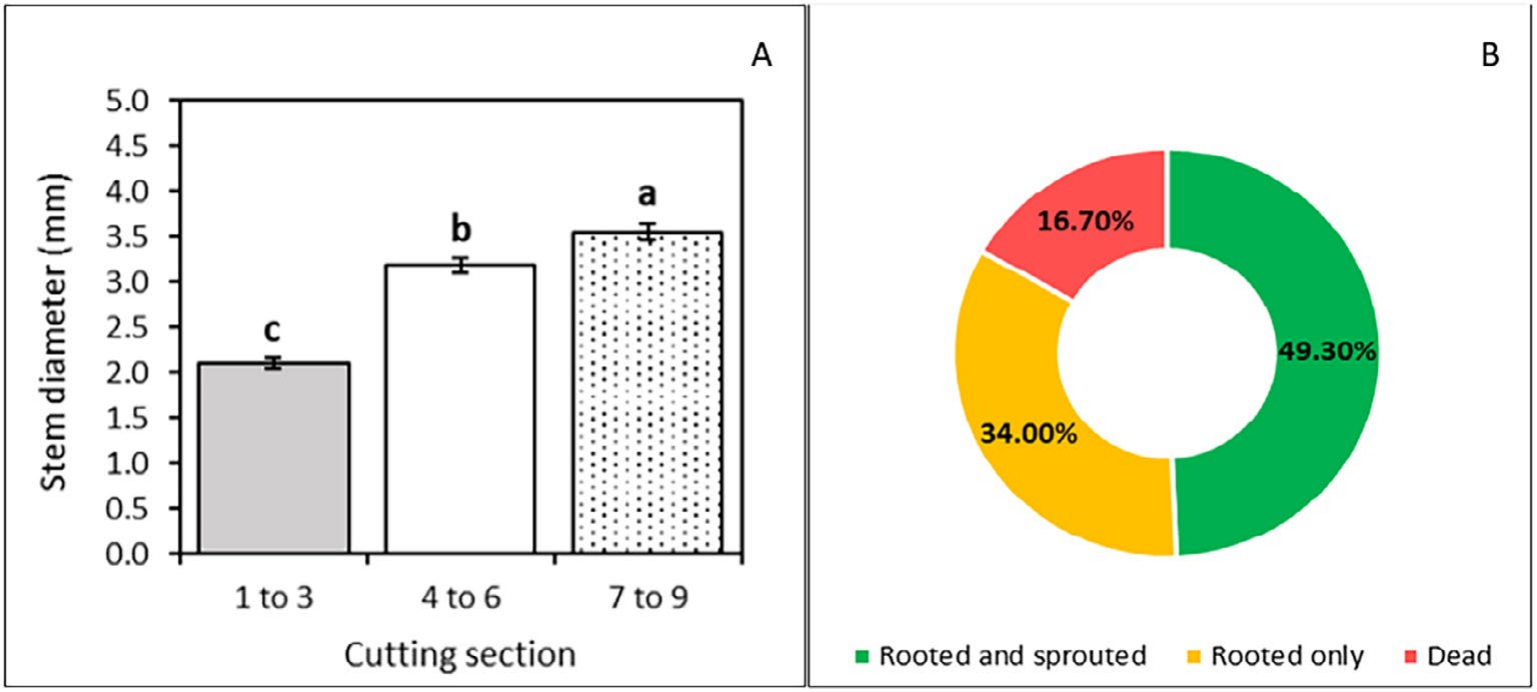
Mean microvine stem diameter ± standard error of the mean in different cutting sections (A) and the proportions of microvine cuttings that rooted and sprouted, rooted only, or died 7 weeks after cuttings were taken (B). Different letters following the means indicate significant differences at a 5% significance level.

The stem diameters of the cutting sections were significantly different (Figure 3A, *p* < 0.05). As expected, the lower sections (nodes 7–9) had the highest average diameter, averaging 3.6 mm, followed by the mid sections (nodes 4–6) with an average of 3.2 mm, and the tip sections (nodes 1–3) with an average diameter of 2.1 mm.

**FIGURE 4:**
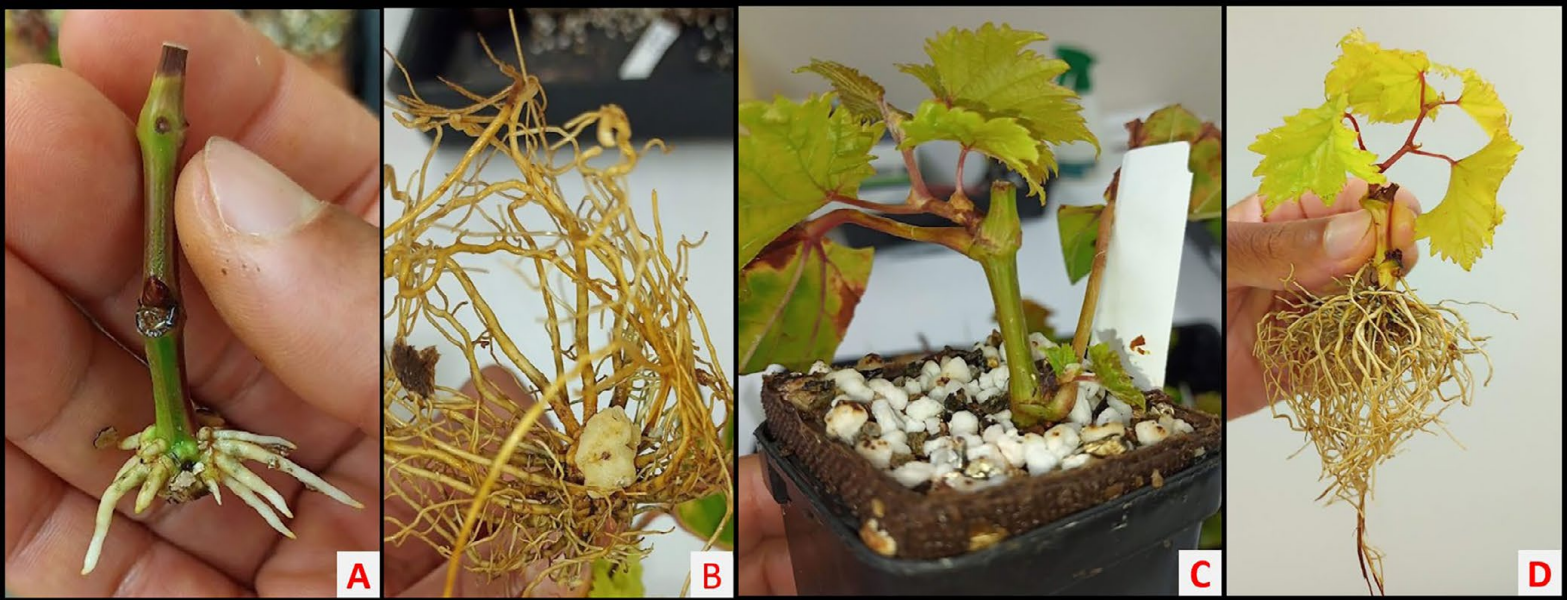
Adventitious roots of microvines formed after 14 days (A), callus (white rounded protuberance) and roots (B), plantlets in vermiculite: Perlite mix (C) and plantlets with roots washed (D) at the end of the propagation experiment (7 weeks).

In total, 83.3% of the cuttings survived at the end of the 7‐week experimental period either with only roots emerged or with both roots and shoots emerged (Figure 3B). Rooting was successful in all surviving cuttings, with the callus and adventitious roots (Figure 4A,B) forming between 14 and 21 days after planting. Bud sprouting started 21 days after planting and continued until the end of the propagation experiment.

While 83.3% of the cuttings formed roots, sprouting success was significantly lower. Average sprouting success across all cutting sections (top, mid, and lower) at 63 days after planting was 49.3% (Figure 3B). A substantial proportion of cuttings (34%) rooted but did not sprout leaves. The cuttings that rooted and sprouted developed into vigorous and healthy plantlets, adequate for immediate transplanting (Figure 4C,D).

#### 3.1.1 Effect of Cutting Section Position and Misting Duration on Bud Sprouting and Cutting Survival

**FIGURE 5:**
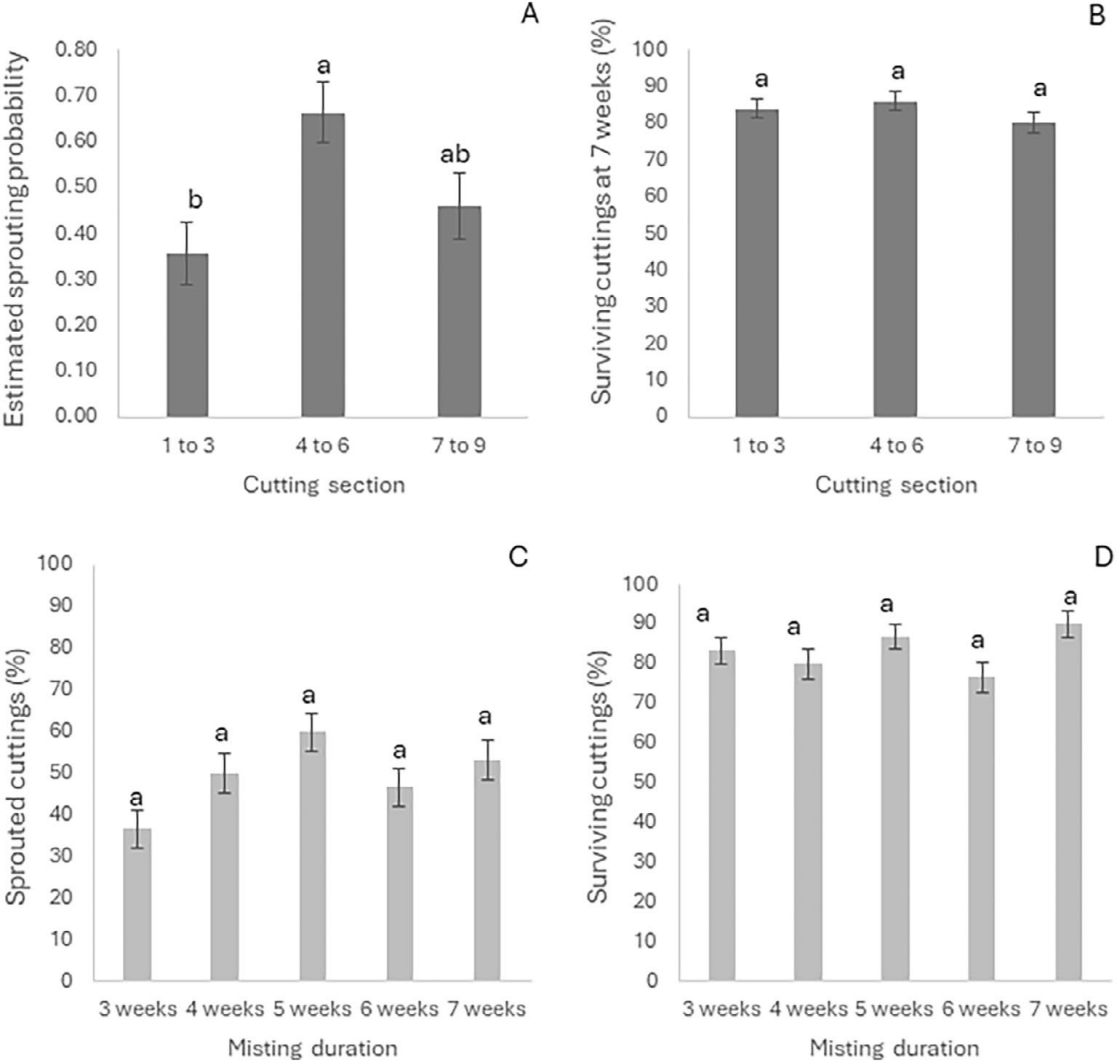
Effect of cutting section position and misting duration on sprouting and survival proportions and probabilities of occurrence. (A) Percentage of cuttings from tip, middle, or lower sections with first leaves sprouted after 7 weeks. (B) Proportion of surviving cuttings (either with only roots emerged or with both roots and shoots emerged) from tip, mid, or lower cane sections after 7 weeks. (C) Proportion of cuttings with first leave sprouted with increasing time under the misting chambers. (D) Proportion of cuttings surviving (either with only roots emerged or with both roots and shoots emerged) with increasing time under the misting chambers.

The cutting sections significantly affected the sprouting success (*p* < 0.05, Figure 5A) but not survival success (Figure 5B). The mid‐section cuttings yielded the highest sprouting success (66%), differing significantly from the tip‐section (36%) but not from the lower section (46%). Similar percentages of cuttings survived after 7 weeks, either with roots only or both roots and shoots emerged, regardless of the cutting section (Figure 5B).

The misting duration, and interaction between misting and cutting sections, did not result in significantly different sprouting and survival success (Figure 5C,D, respectively). However, the 5‐week misting treatment tended to produce the best results, with sprouting proportions of 60% (Figure 5C,D) and survival proportions of 86.7%.

### 3.2 Transplantation and Early Growth Trial

The purpose of the transplantation and early growth trial was to demonstrate that plantlets produced from greenwood cuttings of table grape microvines can be successfully transplanted into a hydroponic growth system and achieve rapid vine growth rates, precocious and continuous flowering and short times to harvest under fully indoor, LED lit conditions.

The logged temperature in the controlled environment room used for the growth experiment remained between 16°C and 26°C throughout the experiment (Figure S2). Relative humidity varied from 57% to 85% with an average of 67%. The light intensity at the top of the canopy varied through the experiment as plants grew closer to the light source. At transplanting the light intensity was 550 μmol.m^−2^.s^−1^ at the top of the pots. On the day before harvest, the measured light intensity at the top of canopy (averaged over 15 point measurements throughout the growth room) was 1147 μmol.m^−2^.s^−1^.

**FIGURE 6:**
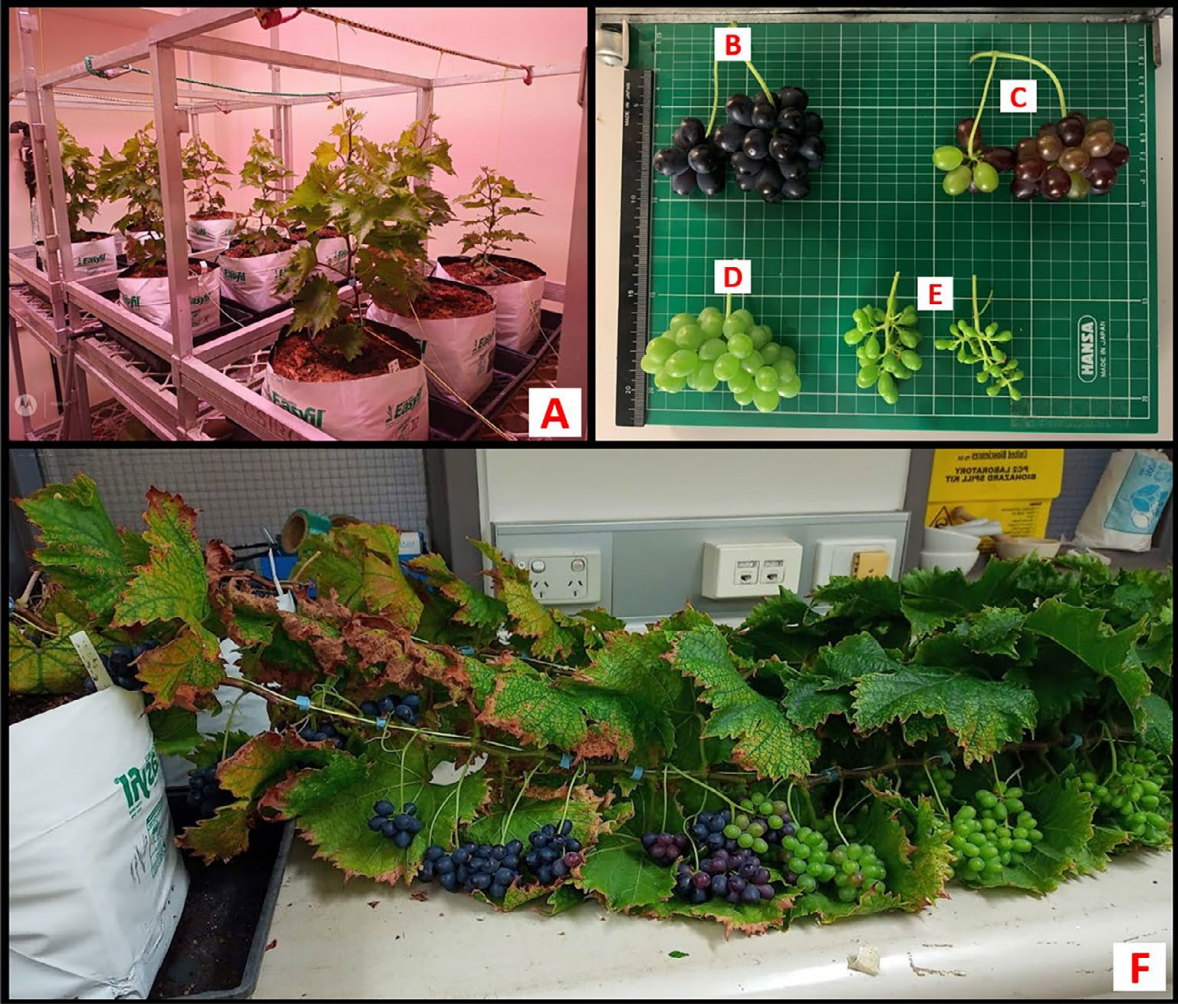
Well‐established microvines (A), fully ripe berries (B), postveraison but still ripening berries (C), preveraison berries (green) (D), and fruit set (E) and fruit load on the microvine main stem (F) at the time of the first harvest.

All the plantlets were established successfully following transplanting (Figure 6A) and survived until the end of the experiment. At harvest (145 days after transplanting), the plants were still undergoing vigorous growth, as demonstrated by formation of new buds and inflorescences in the tip section of the main stem, with the lower and mid sections loaded with fruit clusters (Figure 6F) at different ripening stages (fully ripe, postveraison but still ripening, preveraison or green), and fruit set (Figure 6B–E, respectively).

#### 3.2.1 Effect of Cutting Sections on Establishment, Node (Bud) Formation, Growth, Inflorescence, and Fruit Formation

Mid‐section cuttings took longer to begin forming new nodes and lengthening but had a faster rate of lengthening after onset. As a result, total vine lengths are the end of the experiment were similar between all cutting tissue treatments (more details in Figure S3A,B).

**FIGURE 7:**
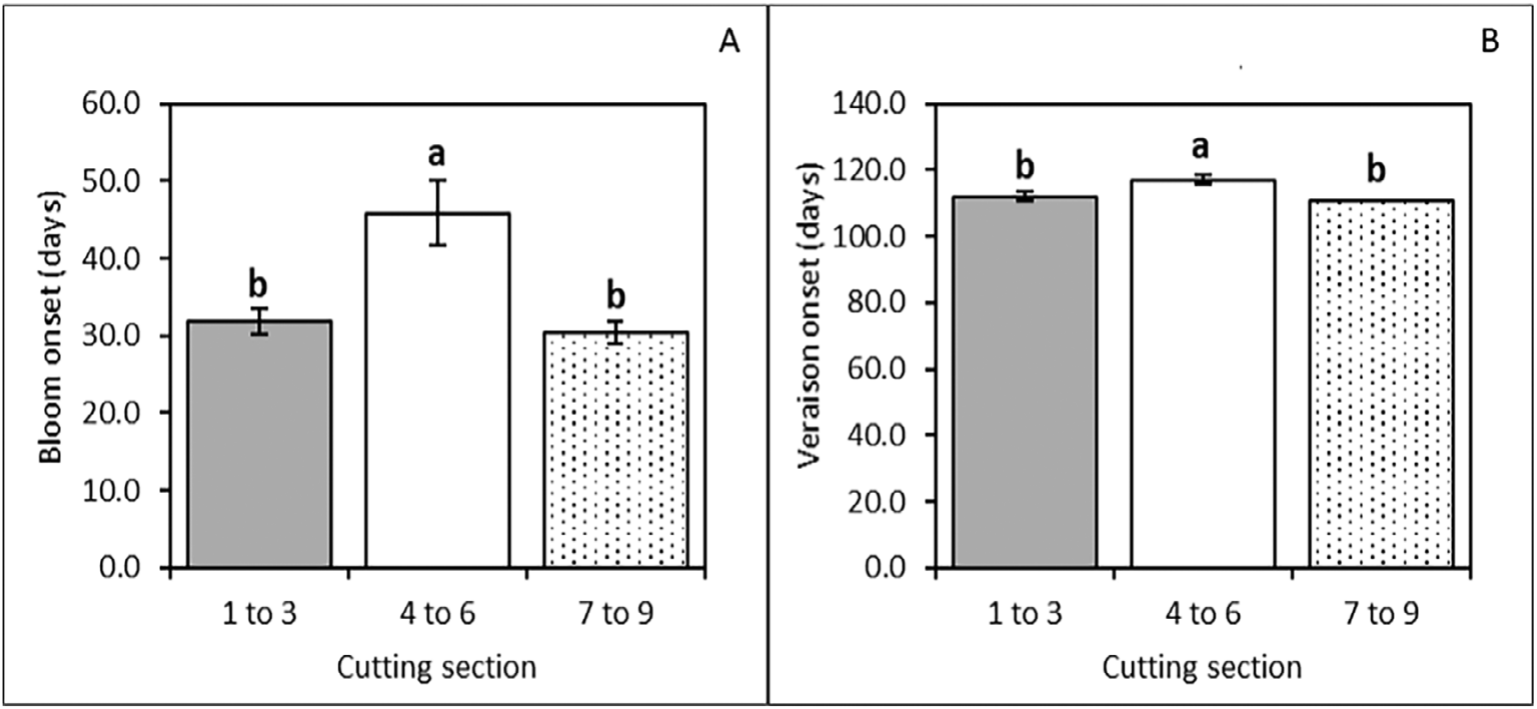
Mean number of days to the onset of flowering (A) and veraison (B) in different cutting sections (±standard error of the mean). Different letters indicate significant differences at a 5% significance level.

Similarly, plants derived from mid‐section cuttings took longer to form the first inflorescences (Figure S4A,B) but the rate of inflorescence formation after onset was faster than plants from tip or lower section cuttings, resulting in similar total inflorescence numbers over the course of the experiment. Vines grown from mid‐section cuttings took slightly longer to reach veraison onset than the vines from tip or lower section cuttings (Figure 7B).

#### 3.2.2 Effect of Cutting Sections on Berry Weight, Yield, Oenology Parameters, and Maturity Index

**FIGURE 8:**
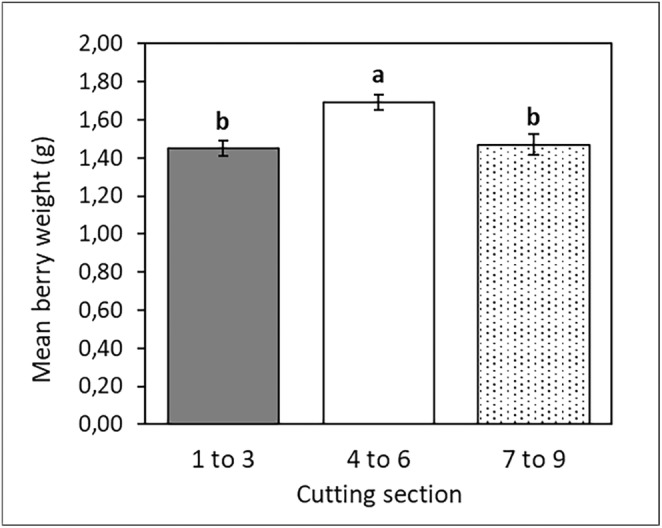
Mean microvine berry weight (± standard error of the mean), as a function of different cutting sections. The same letters indicate means that do not differ significantly at a 5% significance level.

The crop cycle, from when cuttings were taken to first harvest, was 208 days (~7 months), with 63 days for plantlet production and 145 days from transplanting to first harvest. The mean berry weight of ripened berries differed significantly (*p* < 0.05) in cutting source treatments (Figure 8). Vines from the mid‐section cuttings produced the heaviest berries (1.69 g/berry), followed by those from the lower section (1.47 g/berry) and tip cuttings (1.45 g/berry), respectively.

Despite the differences in berry weights, there were no significant differences in the fruit yield per plant or cluster characteristics between the cutting section treatments. The average (± SEM) yield per plant at the time of harvest was 237 (± 38.07) g.plant^−1^. The total number of fruit clusters (including the green clusters) per plant was 20.8 (± 1.59) clusters.plant^−1^ at the end of the experiment. However, it should be noted that all vines had ongoing inflorescence and fruit cluster formation when the experiment was terminated. The average number of berries per cluster, the cluster length, and diameter were, respectively, 12.6 (± 1.29) berries, 4.02 (± 0.206) cm, and 4.97 (± 0.211) cm (Table S1).

**FIGURE 9:**
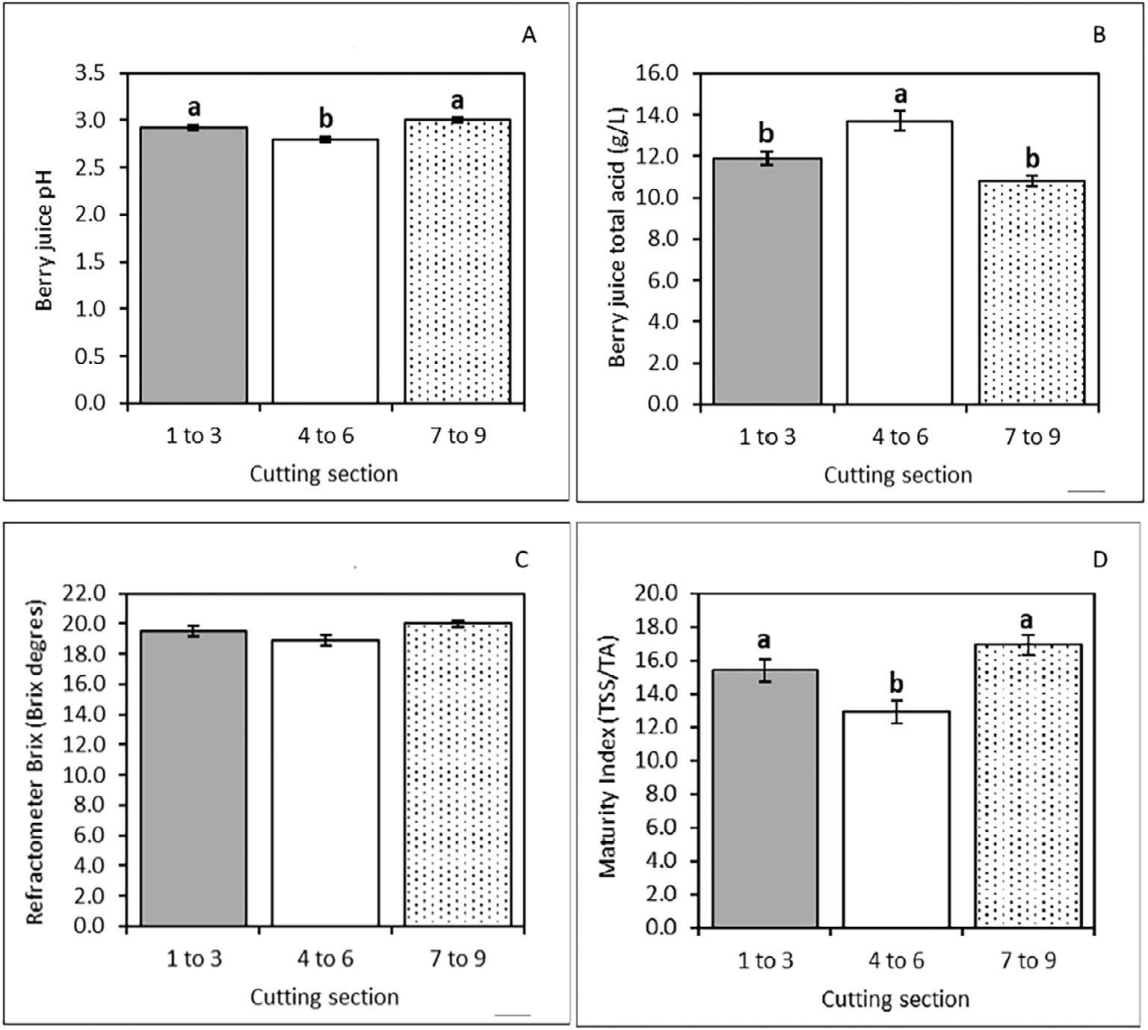
Averages ± standard error of the mean for berry juice pH (A), total acids (B), total soluble solids (^o^Brix) (C), and maturity index (D) of fruit produced from plants derived from different cutting sections. Different letters following the means indicate significant differences at a 5% significance level.

The juice of berries from the vines grown from mid‐section cuttings had significantly lower pH (2.8) and higher total acid concentration (13.7 g.L^−1^) than berries from the vines from tip and lower section cuttings (*p* < 0.05, Figure 9A,B). There were no significant differences between the total soluble solids (^o^Brix) of berries produced under the different treatments, with an overall average (± SEM) of 19.46 (± 0.310) ^o^Brix (Figure 9C). The mid‐section berries also had the lowest maturity index (Figure 9D).

Growing degree days were calculated for the propagation experiment and for the main phenological stages following transplanting of the seedlings (emergence of the first flower, onset of veraison in the first fruit bunch, and harvest (ripeness of the first bunches)) using a base temperature of 10° (Table 3).

**TABLE 3:** Growing degree days for onset of first occurrence of main developmental stages for the grape microvines.

	Measured *t*_max_ (°C)	Measured *t*_min_ (°C)	Growing degree days	Growing days	Cumulative GDD
Propagation	32	15.3	13.65	63	860
First flower	26.5	19.9	13.2	33.9	448
First veraison	26.5	19.9	13.2	116	1534
Transplant to first harvest excluding Propagation	26.5	19.9	13.2	145	1917

## 4 Discussion

This study demonstrates the potential of table grape microvines as a novel crop for intensive, year‐round production in CEF systems. By controlling the growing environment, CEF farmers can increase productivity per unit area, significantly decrease water, fertilizer and pesticide inputs, and escape harmful weather events (O'Sullivan et al. 2020; Benke and Tomkins 2017). As climate change increases risks to field production, the CEF industry is likely to continue to grow as farmers seek ways to protect their crops from variable weather events and changes in flowering, ripening, disease, and pest risks that come with changing climate (Palliotti et al. 2014; Webb, Whetton, and Barlow 2007). However, as the CEF industry scales globally, the industry needs a greater diversity of high‐value crops that are adapted to these systems (O'Sullivan et al. 2019, 2020; Kwon 2023; Kwon et al. 2020).

Previous studies have explored the cultivation of grapevines in hydroponics in both low‐tech CEF structures (tunnel houses) and in highly controlled, LED lit indoor grow rooms (Li et al. 2023; Wheatley et al. 2009; Pisciotta, Barone, and Di Lorenzo 2022). Wild‐type and dwarf grapevines have been grown in hydroponic systems for many years as a research tool as soilless cultivation allows for access to roots and ease of changing nutrient delivery (Dias et al. 2019; Rienth et al. 2012; Torregrosa et al. 2019; Ruperti et al. 2019). Pisciotta, Barone, and Di Lorenzo (2022), note that cultivation of wild‐type grapevines in low‐tech CEF systems has many advantages including opportunities to increase planting density and yield, manipulate vegetative‐reproductive cycles, speed up berry maturity, produce fruit outside traditional harvest seasons, and increase water, fertilizer, and pesticide use efficiencies.

Grape microvines have a range of traits that make them particularly suited to commercial‐intensive production in soilless, CEF systems. Their small stature, indeterminate habit, short juvenile phase, and precocious flowering (flowers produced from every node) mean that they are capable of producing high yields of fruit, year‐round when the growing environment is controlled (Boss and Thomas 2002; Torregrosa et al. 2019). As such, they can be managed in a system that is similar to those used for established greenhouse vine crops such as tomatoes, capsicums, eggplants, and chillies (Raviv and Lieth 2008; Peet and Welles 2005).

### 4.1 Developing Propagation Protocols for Production of Table Grape Microvines Plantlets

The propagation experiment was based on a modification of existing methods for generating grapevine plantlets from green tip cuttings. The experiment tested the use of cuttings collected from three different positions on the shoot (tip, mid, and lower sections) and five durations of misting of the cuttings.

The rooting and survival success of the microvine cuttings were high regardless of the treatments applied. At the end of the 7‐week propagation experiment, 100% of the plantlets had produced roots and more than 80% were alive (Figure 3). Bud emergence was more variable. The high rooting success is likely due to both the appropriate environmental conditions (growth media, misting, light, heating mat, and room temperature) and the use of rooting hormone (IBA). The IBA applied to cuttings is converted into Indole‐3‐acetic acid, a plant hormone that stimulates cell division in the cambium leading to callusing or adventitious roots formation (Frick and Strader 2018; Blythe et al. 2007). Alimam and Agha (2021) found that increasing concentrations of IBA (0, 1.0, and 2.0 g.L^−1^) increased the rooting efficiency from an average of 87.7% (control) to 96.7% in six grape varieties ( *Vitis vinifera* L.). Similarly, Daskalakis et al. (2018), investigating the effects of IBA (0, 0.12, and 0.25 g.L^−1^) and cutting section in four grape varieties, reported an overall increase in rooting from 23% (control) up to 62.7%.

The rooting response to IBA in this study resulted in higher rooting success than reported previously for wild‐type grape vines, that is, 96.7% compared to 62.7% reported by Daskalakis et al. (2018). This may be due to the IBA concentration used (1.5 g.L^−1^ vs. 2 g.L‐1 used by Daskalakis et al. (2018)) but could also reflect the different rooting potential of the microvine variety. Rooted cuttings can absorb water and nutrients from media, increasing the likelihood of survival and allowing the shoot development (Ishtiaq, Haq, and Mohammad 1989; Ram, Pradeep, and Akhilesh 2005), which may explain the finding that all the cuttings that survived were found to have rooted.

While the rooting and survival rates (over 80%) are likely to be acceptable in commercial nurseries, the overall sprouting success after 10 weeks (below 50%, Figure 5) could be either unprofitable to propagators (inefficient system) or unaffordable to producers (expensive plantlets due to high production costs). Previous studies on conventional grapevines reported sprouting rates as high as 83% (Noyce et al. 2022). The high survival rate of unsprouted shoots in this study suggests that it may be possible to improve sprouting success through further studies (Figure 3).

Sprouting success was significantly affected by the cutting tissue source under the propagation conditions used in this experiment, with the mid and basal section cuttings being more likely to sprout than the tip section cuttings (Figure 5). Hansen (1986) found similar results on *Schefflera arboricola* Hayata, where the axillary bud break and shoot growth improved considerably in cuttings from sub‐apical to basal positions. Previous studies on conventional grapevines also reported higher rooting efficiency in cuttings collected from basal sections compared to apical sections (Daskalakis et al. (2018), Keeley et al. (2004), Weaver, Lavee, and Johnson (1975)). Neves, Souza, and De Oliveira (2020) found that the rooting and sprouting in cassava cuttings ( *Manihot esculenta* Crantz) decreased from the apical to basal cuttings with the higher lignification in the basal sections identified as the main driver for their findings. In this study, canes were selected to avoid highly lignified sections, even in the most basal (7–9 node) cuttings, thus, lignification is less likely to have contributed to lower sprouting success in the basal cuttings.

Hartmann et al. (2013) note that root production and shoot growth variability in different cutting sections are common across species, especially in hardwood cuttings, with the best performance frequently observed in the basal sections. The first clues to explain the differential response across cutting sections were established by Tukey and Green (1934) investigating the chemical composition of different cutting sections of *Rosa multiflora* (Thunb.) under different N supply levels. The authors found an increasing gradient in total sugar content, mainly starch, from apical to basal cuttings, with the low nitrogen environment resulting in higher starch contents. This is supported by the recent findings by Bertheloot et al. (2020) on rose plants (*Rosa hybrida* L.) and pea plants ( *Pisum sativum* L.) where increasing levels of sugars, simulated by plant defoliation and external sucrose application, were found to suppress the effects of auxins (apical dominance hormone) and strigolactone (branching inhibitor hormone), stimulating bud outgrowth. Xie, Forney, and Bondada (2018), studying the renewal of vascular connections between grapevine buds and canes during bud break, found that water availability in the bud and the presence of a vascular connection with the cane were fundamental for the sprouting success as these elements enable the circulation of nutrients and hormones. This discussion suggests three lines of inquiry that could be explored to improve sprouting success in microvine cuttings:

- Higher sugar contents may explain the higher sprouting observed in the mid and lower sections in the present study. This hypothesis is supported by the observation of the significantly higher stem diameter in the basal sections (Figure 3A), which correlates with higher carbohydrate reserves (Noyce et al. 2022). Lower sugar levels in cuttings may also explain the overall lower sprouting since cuttings were collected from flowering and fruiting plants. Weyand and Schultz (2006), studying the seasonal dynamics of nitrogen and carbohydrate reserves on conventional grapevine woods (1 and 2 years old and beyond), reported that the minimum concentration of nonstructural carbohydrates was registered between full bloom and bunch closure.
- Moderating N fertilizers on cuttings' parentals (for higher sugar contents in cuttings), efficient irrigation of parental material for hydrated buds (Smart et al. 2002), prior histology analysis (to access vascular continuity in the planting material) and avoiding the apical or lignified planting material could be explored to potentially improve the sprouting success.
- Investigating whether pretreatment with sucrose prior to planting increases the overall sprouting success.

Budburst (sprouting) in the microvine is a complex phenomenon controlled by the interaction of many factors and may differ significantly from wild‐type grapevines, given the differences in plant architecture and lifecycle between the two. In this experiment, no plant growth regulators were applied to stimulate bud burst in the cuttings.

Although the duration of misting treatment did not significantly affect sprouting success, the 5‐week misting treatment resulted in sprouting rates that were almost double that observed with the 3‐week misting treatment (Figure 5). In addition, 3‐week‐old plantlets were inadequate for transplanting (small and fragile leaf apparatus), requiring further growth to achieve viable plantlets after misting was ceased. This suggests that the 5‐week misting treatment is the most favorable propagation duration, as it yields higher sprouting rates and viable plantlets while saving time and costs by avoiding further misting (6 and 7 weeks) without significantly different results in terms of root and shoot emergence and plantlet viability.

### 4.2 Transplantation, Establishment, and Early Growth Demonstration

The purpose of the transplantation and early growth demonstration was to assess the viability and performance of the plantlets produced in the propagation experiment and to demonstrate the growth, flowering, and fruiting of microvines in a fully indoor, LED lit, hydroponic growing system.

All 15 plantlets that were transferred to the hydroponic system, survived, and showed vigorous vegetative and reproductive growth without any symptoms of biotic stress. This demonstrates that if cuttings can root and sprout successfully, the likelihood of successful establishment after transplanting into a hydroponics system is very high. However, this performance was observed under a strictly controlled growth environment, which might not be the case under conventional greenhouses with limited environmental control (Torregrosa et al. 2019) which calls for further experiments to enable the propagation and production of microvines under conventional greenhouses (e.g., natural light, oscillating photoperiod, and temperature).

Despite some differences, the overall performance of vines grown from different cutting sections was similar. Following transplanting into the hydroponic system, the vines grown from mid‐section cuttings had a slightly longer lag before the onset of stem elongation and new node formation compared to vines from the tip and lower section cuttings (Figure S3). This suggests that mid‐section cuttings had a delayed establishment which was later translated into delayed initiation in the onset of inflorescence and fruit cluster formation (Figure S4) and consequently veraison (Figure 7). However, the mid‐section had the fastest rates of node and inflorescence formation, and stem elongation. This advantage allowed the mid‐section plantlets to catch up and reach the same results as other treatments for most of the parameters measured on the harvesting day, including yield components (Figure 9 and Table S1). The only remaining difference was the berry maturation parameters (Figure 9), with berries from mid‐section cutting vines slightly less mature than those from tip or lower section cuttings.

This suggests that, despite the initial differences Post‐transplanting, the plantlets have the same development and reproductive potential regardless of their source (cutting section). Considering this, the mid‐section planting material is more desirable, as it yielded the highest sprouting rate while giving a similar performance after transplantation. The values for berry weight, Brix, and internode length registered in the present study were slightly higher compared to those reported by Luchaire et al. (2013) and Rienth et al. (2012), although the number of berries per cluster was lower than those reported in the previous studies, suggesting that the experimental environment was appropriate for the microvines. The lower berry counts may be due to the relatively young age of the vines at the time of the harvest in this experiment.

The GDD to harvest of 1917 degree days is categorized as having potential for quality production for most varieties of traditional grapevines under outdoor conditions (Jarvis and Englefield 2018). However, this result should be read with some caution because it is difficult to apply the concept of GDD directly to microvines grown under controlled environment conditions. Microvine's indeterminate habit and continuous fruiting mean that all EL stages are occurring on any given vine at the same time after the first harvest (e.g., lower nodes will hold ripe fruit ready for harvest while nodes higher up the vine will be in flowers, and nodes closer to the vine tip will be at bud‐burst). Here, the GDD has been calculated only for the lowest nodes of the vines (i.e., the first bunches to form and ripen) to give an indication of the heat accumulation required for the full development of the fruit on the microvines. Additionally, the use of a full‐controlled environment means that both the temperature and day length could be altered to speed up or delay fruit development and ripening as required.

It is difficult to estimate the annual yield of the vines under the experimental conditions because of the short experimental timeframe combined with the indeterminate and continuous flowering habit of the vines. The observed average yield per plant (237 g), represents only the first harvest, where only 30%–40% of the total clusters on the vines present at that time were harvested. This is likely to be a small fraction of the total annual productivity of the vines. First, because the bunches harvested are likely to be smaller than those that will form as the vines mature, and second, because it is not known if the growth rate observed during this establishment and early growth period will be stable as the vines mature. It is possible that the growth rate, berry count per cluster, berry weight, and cluster formation rates could either speed up or slow down as the vines continue to grow. These factors could also be influenced by management practices including pruning, training, and plant nutrition. Further studies are needed to fully explore the productivity of table grape microvines in CEF and to optimize their management to maximize yield and fruit quality.

It is possible to calculate a rough estimate of the potential productivity of the microvines grown in CEF based on the data gathered in this early growth trial. Considering that it took 74 days from the first berry formation to the first harvesting (data not presented), a minimum of five harvests.year^−1^ with a berry yield of 1.25 kg.plant^−1^.year^−1^ can be estimated. This is at the lower end of the estimates reported by Pisciotta et al. (2022) for wild‐type grapevines grown in a soilless system. However, microvines have two advantages over wild‐type vines. First, they begin flowering and fruiting much earlier. In this case, the first ripe bunches were produced in ~7 months compared to 2–3 years for field‐grown, wild‐type grape vines. Second, they can be planted at higher densities than those reported by Pisciotta et al. (2022). In this study, 15 plants were grown in a ~6 m^2^ room (~25,000 plants.ha^−1^), and this planting density could yield 31.25 tons.ha^−1^.year^−1^. This planting density is similar to those reported for commercial production of other vine crops grown in greenhouses (Peet and Welles 2005). It should be noted that previous measurements of productivity during the breeding of these vines have achieved significantly higher yields per plant, up to 3 kg.plant^−1^.year^−1^ (pers comm, Ian Dry). Both the planting density and the significantly shortened time to first harvest, make this system likely to be more productive per unit area than CEF production of wild‐type grapevines. However, longer experiments are required to verify the yields measured in this trial and to optimize yields from microvines grown in CEF.

The ripened berries from all cutting sections presented a suitable maturation standard for the Australian market, with an average Brix of 19.46 ^o^Brix, without significant differences between treatments. According to the Biosecurity and Agriculture Management (Agriculture Standards) Regulations 2013, the minimum standard of maturity (2020/2021) for standard table grapes varieties ranged between 15 and 18.5 ^o^Brix. However, due to the delayed onset of veraison of grapes on mid‐section cuttings had a significantly lower maturity index (ratio between TSS and total acids, Figure 9E) at the harvest date, as explained by the higher total acid content at harvesting (Figure 9B), especially the Malic acid (data not shown). This corroborates with Moreno and Peinado (2012), who indicate that 90% of grape acidity at maturation is due to malic acid.

## 5 Conclusions

This study demonstrates the potential of table grape microvines as a novel crop for controlled environment farms. The experiments also generated data to inform the development of microvine propagation protocols, a critical first step for the successful integration of microvines in the CEF market. The results showed that the rooting and survival success of microvines is high, with 100% rooting and above 80% survival of cuttings after 7 weeks. However, the sprouting success is lower, at an average of 49.3%. The use of mid and lower cutting sections in combination with a 5‐week misting treatment resulted in the highest levels of sprouting.

The transplantation and early growth trial showed that, if cuttings can be rooted and sprouted, the likelihood of successful establishment in a hydroponic system is high regardless of the plantlets' source (cutting section). Although the mid‐section plantlets showed delayed establishment and onset of veraison, the overall production potential and fruit quality are similar in all cutting sections.

Further research is needed to improve the sprouting success of microvines to the potential 80% and to verify the productivity of table grape microvines in CEF. Potential research areas to improve sprouting could include the effects of sucrose treatment on cuttings, conventional glasshouse, and optimizing parental material growth environments and timing of cutting collection.

## Conflicts of Interest

The authors declare no conflicts of interest.

## Supporting information


Data S1.

## Data Availability

The data that supports the findings of this study are available in the Supporting Information of this article.
