# Supplementary material for: Propagation, Establishment, and Early Fruit Production of Table Grape Microvines in an LED‐Lit Hydroponics System: A Demonstration Case Study
Source: Plant Environ Interact. 2024 Dec 1;5(6):e70018. doi: 10.1002/pei3.70018 (PMC11608432; doi:10.1002/pei3.70018)
Supplement: Supplementary file 1 — Data S1. [file PEI3-5-e70018-s001.docx]

**Figure S1:** Average temperature (^o^C) and relative humidity (%) under plastic domes during the propagation experiment.

**Figure S2:** Average temperature (^o^C) and relative humidity (%) the controlled growth room throughout the transplantation and growth experiment.

**Figure S3:** Node formation (A) and main stem elongation (B) over time as a function of cutting sections. The bi-linear model equation is Y = a + b*(Days – xs). The change in angle of the trend line indicates the time in days of onset of node formation (A) and stem elongation (B).


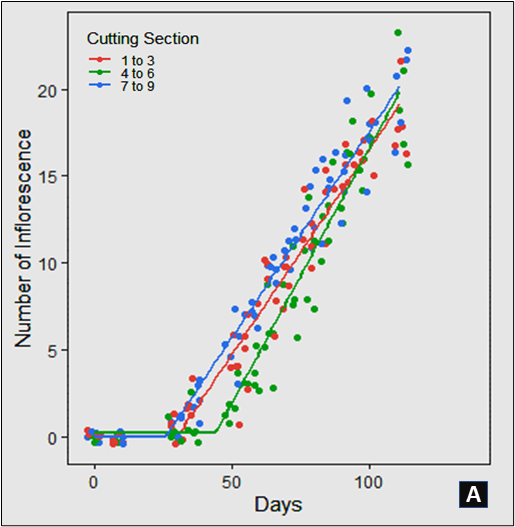

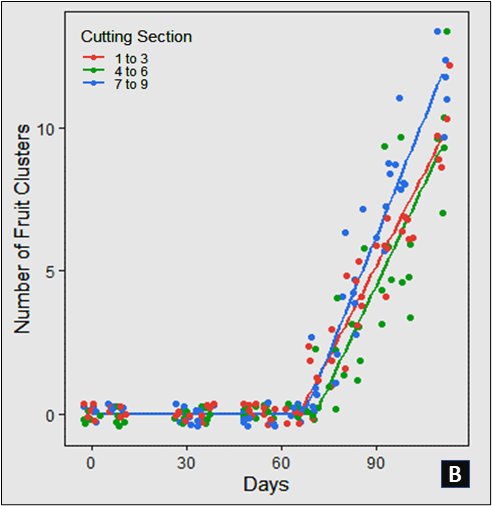

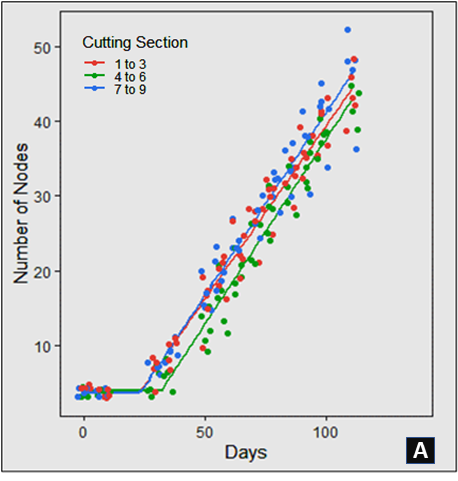

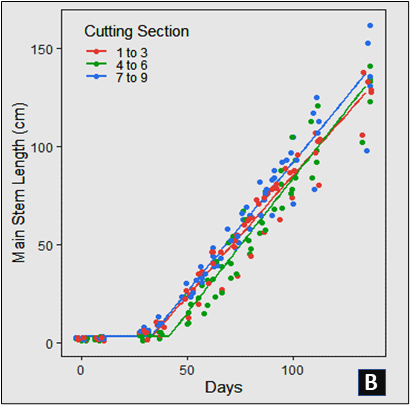


**Figure S4:** Number of inflorescences (A) and fruit clusters (B) produced over time, as a function of different cutting sections. The bi-linear model equation is *Y = a + b*(Days – xs)*. The change in angle of the trend line indicates the time in days of onset of inflorescence formation (A) and fruit cluster formation (B).

**Table S1:** Descriptive statistics of the variables measured on the harvesting day.

| **Descriptives** | **Cutting section** | **Plant height (cm)** | **Main stem diameter (mm)** | **Internode length (cm)** | **Nodes per plant** | **Ripen clusters per plant** | **Total clusters per plant** | **Yield (g/plant)** | **Berries per cluster** | **Cluster length (cm)** | **Cluster diameter (cm)** |
| --- | --- | --- | --- | --- | --- | --- | --- | --- | --- | --- | --- |
| Mean | 4 to 6 | 161 | 0.992 | 3.10 | 61.0 | 9.00 | 21.0 | 242.0 | 12.1 | 3.98 | 5.13 |
|  | 7 to 9 | 170 | 0.960 | 2.92 | 64.4 | 10.2 | 20.4 | 234.0 | 11.8 | 4.01 | 4.89 |
|  | 1 to 3 | 151 | 0.926 | 2.86 | 60.4 | 8.00 | 21.0 | 235.0 | 14.0 | 4.05 | 4.89 |
| **Overall mean** | | 160.67 | 0.96 | 2.96 | 61.93 | 9.07 | 20.80 | 237.0 | 12.63 | 4.01 | 4.97 |
| Std. error mean | 4 to 6 | 8.50 | 0.0335 | 0.0765 | 2.45 | 0.837 | 1.52 | 46.1 | 1.47 | 0.216 | 0.215 |
|  | 7 to 9 | 12.5 | 0.0358 | 0.0934 | 3.85 | 0.860 | 2.69 | 31.1 | 1.19 | 0.217 | 0.210 |
|  | 1 to 3 | 5.54 | 0.0376 | 0.0763 | 1.89 | 0.837 | 0.548 | 37.0 | 1.21 | 0.186 | 0.208 |
| **Overall Std. error mean** | | 8.84 | 0.0356 | 0.0821 | 2.73 | 0.845 | 1.59 | 38.07 | 1.29 | 0.206 | 0.211 |
| Minimum | 4 to 6 | 137 | 0.870 | 2.89 | 52.0 | 7.00 | 18.0 | 142.0 | 8.99 | 3.44 | 4.42 |
|  | 7 to 9 | 122 | 0.820 | 2.58 | 51.0 | 7.00 | 11.0 | 162.0 | 9.59 | 3.40 | 4.43 |
|  | 1 to 3 | 137 | 0.780 | 2.69 | 55.0 | 6.00 | 20.0 | 118.0 | 10.0 | 3.59 | 4.08 |
| Maximum | 4 to 6 | 184 | 1.05 | 3.31 | 67.0 | 12.0 | 26.0 | 391.0 | 16.1 | 4.57 | 5.62 |
|  | 7 to 9 | 191 | 1.02 | 3.15 | 75.0 | 12.0 | 26.0 | 333.0 | 16.1 | 4.70 | 5.53 |
|  | 1 to 3 | 167 | 0.980 | 3.13 | 65.0 | 10.0 | 23.0 | 341.0 | 17.3 | 4.47 | 5.25 |
